# Key influence of sex on urine volume and osmolality

**DOI:** 10.1186/s13293-016-0063-0

**Published:** 2016-02-09

**Authors:** Majuran Perinpam, Erin B. Ware, Jennifer A. Smith, Stephen T. Turner, Sharon L. R. Kardia, John C. Lieske

**Affiliations:** Division of Nephrology and Hypertension, Mayo Clinic, 200 First Street SW, Rochester, MN 55905 USA; Institute for Social Research, University of Michigan, Ann Arbor, MI USA; Department of Epidemiology, School of Public Health, University of Michigan, Ann Arbor, MI USA; Department of Laboratory Medicine and Pathology, Mayo Clinic, Rochester, MN USA

**Keywords:** Urine osmolality, Diet, Nephrolithiasis, Urine volume

## Abstract

**Background:**

Demographics influence kidney stone risk and the type of stone that is more likely to form. Common kidney stone risk factors include having a low urine volume and a high urine concentration. The goal of the current study was to evaluate the effect of demographics on urinary concentration and osmole excretion.

**Methods:**

Twenty-four-hour urine samples were collected from non-Hispanic white sibships in Rochester, MN. Height, weight, blood pressure, serum creatinine, and cystatin C were measured. Diet was assessed using the Viocare food frequency questionnaire. Effects of demographics and dietary elements on urine osmolality and volume were evaluated in bivariate and multivariable models, as well as models that included dietary interactions with age, sex, and weight.

**Results:**

Samples were available from 709 individuals (mean age 66 ± 9 years, 59 % female). Across the age spectrum, males had higher urine osmolality (~140 mOsm/kg, *p* < 0.0001) and total osmole excretion (~270 mOsm, *p* < 0.0001) compared to females. For any given urine volume, males had a consistently higher urine osmolality (~140 mOsm/kg, *p* < 0.0001). In multivariable models, urine osmolality declined with age and water intake and remained higher in males than females. Urine osmolality positively associated with weight and animal protein intake. Higher urine volume associated with larger water intake. An interaction revealed that greater body weight was associated with larger changes in urine osmolality as oxalate intake increased (*p* = 0.04).

**Conclusion:**

Data from this study support the hypothesis that there are sex differences in thirst and vasopressin action. This trend in urine concentration is also consistent with known epidemiologic patterns of urinary stone disease risk.

**Electronic supplementary material:**

The online version of this article (doi:10.1186/s13293-016-0063-0) contains supplementary material, which is available to authorized users.

## Background

Kidney stones are common with up to 10 % of people experiencing one during their lifetime [1]. Furthermore, up to 50 % of stone formers will recur within 5 years of their first stone [1]. Human urine is almost always supersaturated for one or more crystal types that can form stones (i.e., calcium oxalate, calcium phosphate, and uric acid). High fluid intake has been universally advocated for stone prevention in order to favor more dilute urine. Thus, recent guidelines from the American Urological Association (AUA) and American College of Physicians (ACP) both recommend sufficient fluid intake to maintain urine volume of 2.0 to 2.5 L [2, 3]. Therefore, urine osmolality and volume are relevant factors to assess in the context of kidney stone risk.

Demographics are known to influence kidney stone risk and even the type of stone that is more likely to form [4]. For example, kidney stones are more common in males, obese subjects, and those less than 70 years old [4, 5]. However, the effects of these factors on key urine characteristics that associate with stone risk have not been carefully examined. The key regulators of urinary concentration and volume are blood vasopressin levels and thirst. Minimal data suggest sex can influence one or both factors [6, 7]. Thus, the goal of the current study was to evaluate the effect of demographics (including sex) and diet on urinary concentration and osmole excretion. To do so, we took advantage of data from a large cohort of well-characterized subjects for whom complete urinary stone risk profiles were available.

## Methods

This study was approved by the Mayo Clinic Institutional Review Board.

### GENOA cohort

The multi-phase Genetic Epidemiology Network of Arteriopathy (GENOA), a member of the Family Blood Pressure Program (FBPP), recruited non-Hispanic white hypertensive sibships from Rochester, Minnesota (MN), for linkage and association studies to investigate the genetic underpinnings of hypertension in phase I (1996–2001) [8]. The Genetic Determinants of Urinary Lithogenicity (GDUL) study (2006–2012) is an ancillary study conducted in Rochester, MN, GENOA cohort members [9]. Participants were invited to collect 24-h urine samples and complete a food frequency questionaire (FFQ, Viocare Technologies, Princeton, NJ, USA) [10]. Participants were excluded from this study if they were in endstage renal failure (stage 5 CKD). All other GENOA subjects were eligible. Of note, recruitment for the original GENOA study and the current GDUL ancillary study was not based on CKD status or on the presence (or absence) of urinary stones.

### Study visit

After informed consent, participants completed at least one 24-h urine collection [11, 12] and the FFQ at a CKD and/or GDUL study visit. A total of 299 (42.7 %), 227 (32.0 %), and 183 (25.8 %) participants had a total of one, two, or three urine collections, respectively. For individuals with two or three urine collections, values were averaged for analysis. The mean time between the earliest and latest urine collections was 1.73 years (range = 0.9 to 3.6 years). The average time between the two GDUL collections was 22 days. Intraclass correlation coefficients (ICCs) for urine factors across collections revealed that the majority of urine measures were relatively stable across time. Urine osmolality ICC was 0.59 and urine volume ICC was 0.67. Participants also completed a detailed Kidney Stone Questionnaire (to assess stone forming status). Subjects completed the questionnaires at the time of a study visit, which was in general within 1 to 2 days of the urine collection.

### Urine collection

Toluene (30 ml) was added as a preservative [13] to the collection bottle at the start of all 24-h collections.

Twenty-four-hour urine osmolality, volume, sodium, and potassium were measured in the Mayo Clinic Renal Testing Laboratory. Serum creatinine was assessed using a standardized enzymatic assay on a Roche Cobas chemistry analyzer (c311) (Roche Diagnostics; Indianapolis, IN, USA) while cystatin C was measured using an immunoturbidimetric assay (Gentian; Moss, Norway) that was traceable to an international reference material. Glomerular filtration rate (GFR) was independently estimated using cystatin C (eGFR_Cys_) [14].

### Descriptive statistics

Data management and statistical analyses were conducted in SAS version 9.3 (SAS Institute Inc., Cary, NC, USA) [15]. Urine measures appeared to have relatively normal distributions; thus, no variable transformations were applied. Values that were ≥4 standard deviations from the mean of any urine or diet measure were removed. The contribution of electrolytes to urine osmole load was estimated as 2 × (urine sodium + urine potassium), while urea contribution was calculated as the difference between the total osmole excretion and electrolyte contribution. Linear mixed effects models (LMM) that included sibship as a random intercept (to properly account for family structure) were used to test whether there were significant differences by sex for the urinary and diet measures.

### Association testing

To account for the sibships, a randomly selected, independent subset of the GENOA cohort (one individual per sibship; *n* = 414) was used for stepwise linear regression to determine the variables that were associated with each urinary measure. Variables available for selection included the following: weight, body mass index (BMI), smoking status (current or never smoker), diabetes status (yes/no), fasting blood glucose level, systolic blood pressure (SBP), diastolic blood pressure (DBP), eGFR_Cys_, diuretic loop use (yes/no), diuretic thiazide use (yes/no), and dietary variables from the FFQ including animal protein, sodium, water (including food-derived water), calcium, fructose, oxalate, total protein, and sucrose intakes. The entry criterion was *p* < 0.05, and the exit criterion was *p* > 0.10. Age, sex, and serum creatinine were forced into each model.

After model selection, LMM was performed on the full GENOA sample to assess significant predictors of the urinary measures, accounting for the sibship structure in GENOA. Interaction models were also conducted to assess interactions of age, sex, and weight (if weight was included in the model selection as a predictor) with the variables included in the models. Interactions were considered significant at an alpha level of 0.05.

**Fig. 1:**
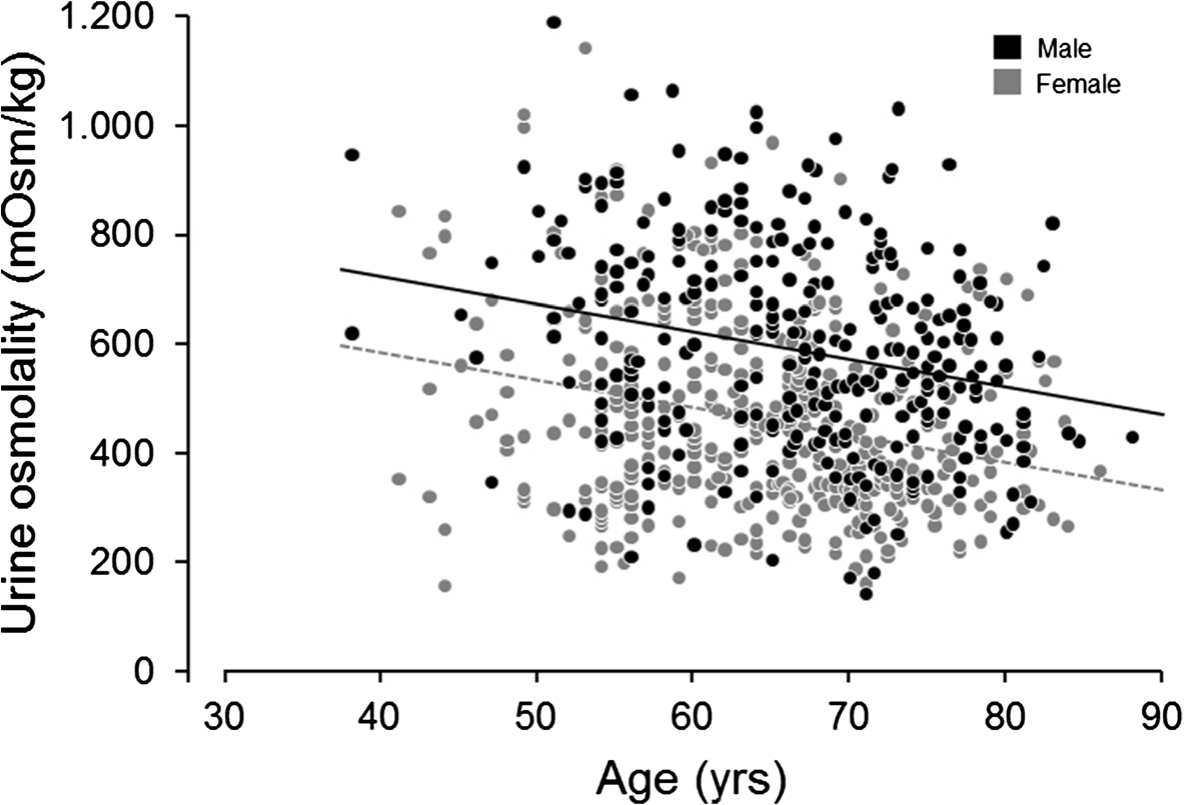
Effect of age on urine osmolality in males and females (age *β* = −5.00, *p* < 0.0001; sex *β* = 142.6, *p* < 0.0001)

**Fig. 2:**
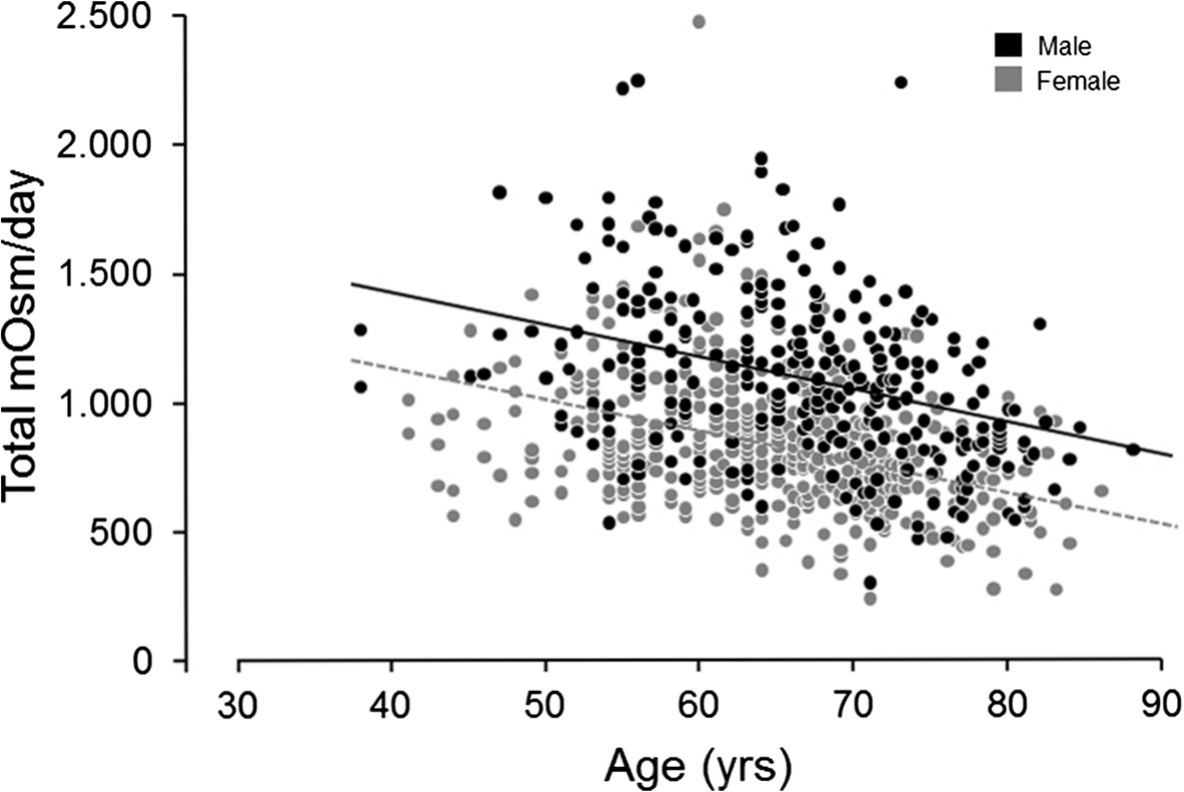
Relationship between total urine osmole excretion and age in females and males (age *β* = −12.296, *p* < 0.0001; sex *β* = 272.633, *p* < 0.0001)

**Fig. 3:**
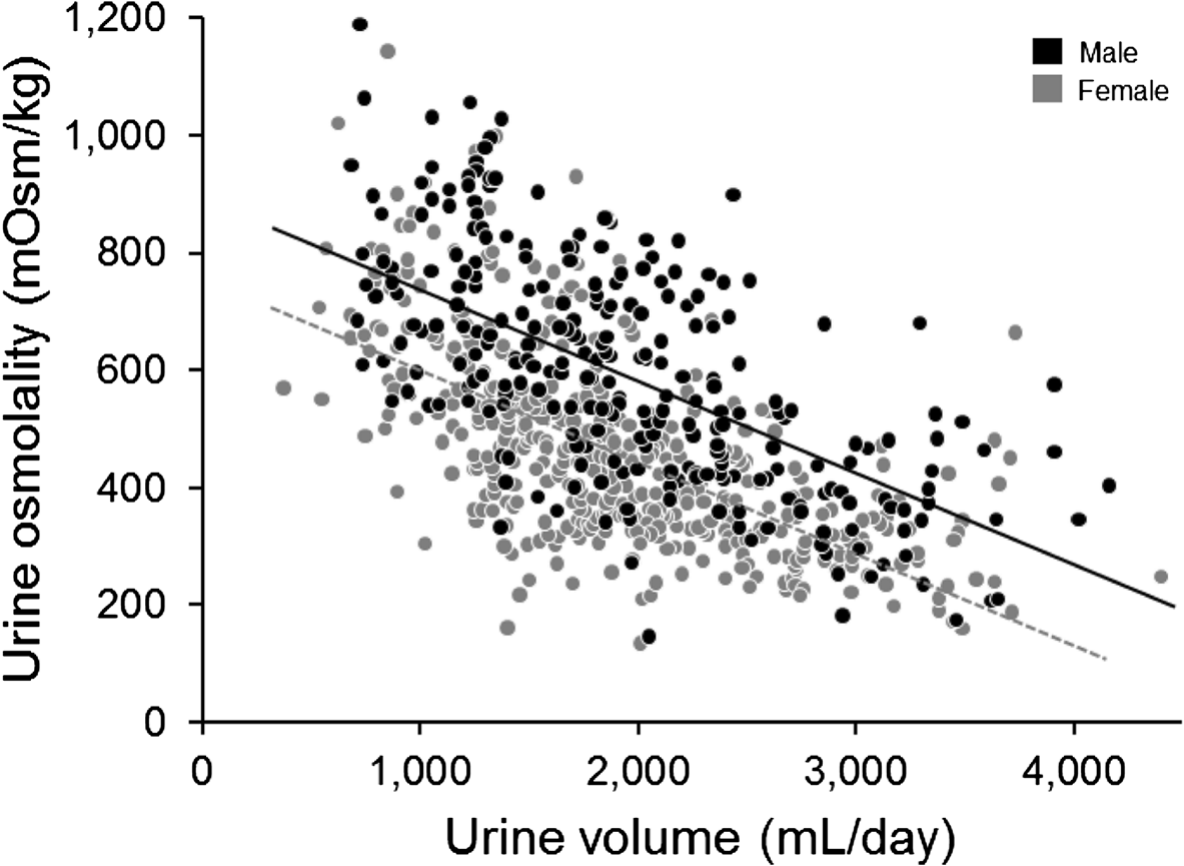
Relationship between urine osmolality and volume in females and males (volume *β* = −0.1597, *p* < 0.0001; sex *β* = 135.63, *p* < 0.0001)

Figures 1, 2, and 3 were created using a scatter plot of the variable of interest (age or urine volume) and an outcome variable (urine osmolality or total mOsm/day) to visualize the relationship between the two variables. Scatter plots were colored by gender, and linear mixed model regression lines were superimposed on the scatter plots controlling for sex and accounting for sibship structure. Lines were plotted by taking the intercept for males and the intercept for females, with the slope from the variable of interest. The beta estimate for sex is reported as the difference in outcome variable for males versus females with corresponding significance.

## Results

A total of 709 individuals from 414 sibships participated in this study (Table 1). The sibship structure of the sample was as follows: 211 singletons, 148 sibpairs, 35 sibships with 3 siblings, and 20 sibships with 4 or more siblings. The mean age was 66 ± 9 years and 59 % of the participants were female. Out of 709 participating individuals, 577 provided information on kidney stone history, of whom 67 (overall 11.6 %; 35 men (14.8 %) and 32 women (9.4 %)) had had a previous stone, reflecting urinary stone disease prevalence in the general population [3]. Three individuals were on medications for stone prevention (potassium citrate). A minority were in CKD stage 3 (10.1 %) or stage 4 (0.5 %). Use of medications that alter the renin-angiotensin system was similar in men (48.5 %) and women (41.8 %).

**Table 1:** Descriptive statistics

		Combined	Female	Male	
			*n* = 416	*n* = 293	
	*n*	Mean (SD) or *n* (%)	Mean (SD) or *n* (%)	Mean (SD) or *n* (%)	*p* value
Age, years	709	65.4 (9)	64.6 (8.9)	66.5 (9)	0.05
Weight, kg	709	87.7 (19.1)	81.3 (17.5)	96.8 (17.5)	<.0001
BMI, kg/m^2^	709	31 (5.9)	30.9 (6.5)	31 (5)	1.00
SBP, mmHg	705	149 (25)	150 (25)	147 (25)	0.09
DBP, mmHg	705	84 (11)	82 (11)	86 (11)	0.95
Serum creatinine, mg/dL	612	0.9 (0.2)	0.8 (0.2)	1.0 (0.2)	<.0001
eGFR_Cys_, ml/min/1.73 m^2^	601	85.6 (24.7)	87.9 (25.8)	82.5 (22.8)	0.02
Diabetes status	625				0.21
Yes		87 (13.9)	45 (12.4)	42 (16.1)	
No		538 (86.1)	319 (87.6)	219 (83.9)	
Blood glucose, mg/dL	612	96.1 (22.9)	94.8 (23.0)	98.0 (22.7)	0.24
Dietary measures					
Oxalate, mg/day	511	215.8 (126.3)	217.9 (121.3)	212.9 (133.3)	0.64
Animal protein, g/day	521	52.9 (25.4)	48.9 (21.4)	58.7 (29.4)	<.0001
Sodium, mg/day	521	3140 (1407)	2947 (1313)	3419 (1491)	<0.0001
Water intake, g	521	2950 (1119)	3004 (1050)	2873 (1211)	<0.0001
Total protein, g/day	521	80.4 (33.9)	75.5 (29.9)	87.3 (37.9)	<0.0001
Sucrose, g/day	519	37.2 (20.7)	38 (20.4)	36 (21.1)	0.34
Calcium, mg/day	521	1059 (542)	1060 (517)	1057 (578)	<0.0001
Diuretic use					
Loop	709				0.61
Yes		35 (4.9)	22 (5.3)	13 (4.4)	
No		674 (95.1)	394 (94.7)	280 (95.6)	
Thiazide	709				0.99
Yes		259 (36.5)	152 (36.5)	107 (36.5)	
No		450 (63.5)	264 (63.5)	186 (63.5)	
Urinary traits					
Urine osmolality, mOsm/kg	709	511.3 (188.6)	456.1 (165.7)	589.8 (191.7)	<0.0001
Urine volume, mL/day	709	1971.1 (690.77)	1967.65 (675.26)	1975.99 (713.36)	0.8743
Total mOsm/day	709	932 (314)	829 (257)	1078 (329)	<0.0001
Urine sodium, mmol/day	705	143 (58)	123 (48)	170 (61)	<0.0001
Urine potassium, mmol/day	709	59 (23)	52 (19)	70 (23)	<0.0001
Electrolyte contribution to urine osmole load, mmol/day	705	403 (145)	349 (116)	478 (148)	<0.0001
Urea contribution to urine osmole load, mmol/day	705	523 (242)	476 (212)	591 (264)	<0.0001

*p* values were testing for sex differences, using linear mixed models to account for sibships. Water intake includes water from food consumption

*SD* standard deviation, *BMI* body mass index, *SBP* systolic blood pressure, *DBP* diastolic blood pressure, *eGFR* estimated glomerular filtration rate (cystatin calculation)

In the bivariate analysis (Table 2), increased urine osmolality significantly associated with decreased age and water intake; male sex; and increased serum creatinine, weight, dietary animal protein, and sodium intake (*p* values all <0.05). Increased urine volume significantly associated with lower age and serum creatinine, and higher dietary animal protein, oxalate, sodium, and water intake (*p* values all <0.05). Across the age spectrum, males had a roughly 140 mOsm/kg higher urine osmolality (*p* < 0.0001) and approximately 270 mOsm higher total osmole excretion (*p* < 0.0001) compared to females (Figs. 1 and 2). Thus, males also had a higher average osmolality (~140 mOsm/kg, *p* < 0.0001) after accounting for urine volume (Fig. 3). Men also had a greater osmole excretion than women (1078 vs 829 mOsm/day) (Table 1). This was due to roughly equal contributions of greater excretions of electrolytes (478 vs 349 mmol/day) and urea (591 vs 476 mmol/day) in men compared to women. Variance in urine volume and osmolality did not significantly differ between the sexes (see Additional file 1: Figure S1).

**Table 2:** Bivariate associations for urine osmolality and volume

	Urine osmolality, mOsm/kg	Urine volume, mL/day
	*β*	*β*
Age, years	−4.19***	−9.68**
Sex (male)	134.10***	8.84
Serum Creatinine, mg/dL	107.95**	−334.26**
Weight, kg	3.58***	0.66
Dietary measures		
Animal protein, g/day	1.51***	2.40*
Oxalate, mg/day	−0.11	0.77**
Sodium, mg/day	0.018**	0.062**
Water Intake, g	−0.031***	0.21***

Water intake includes water from food consumption

*β* beta estimate

*p value <0.05; **p value <0.01; ***p value <0.001

**Fig. 4:**
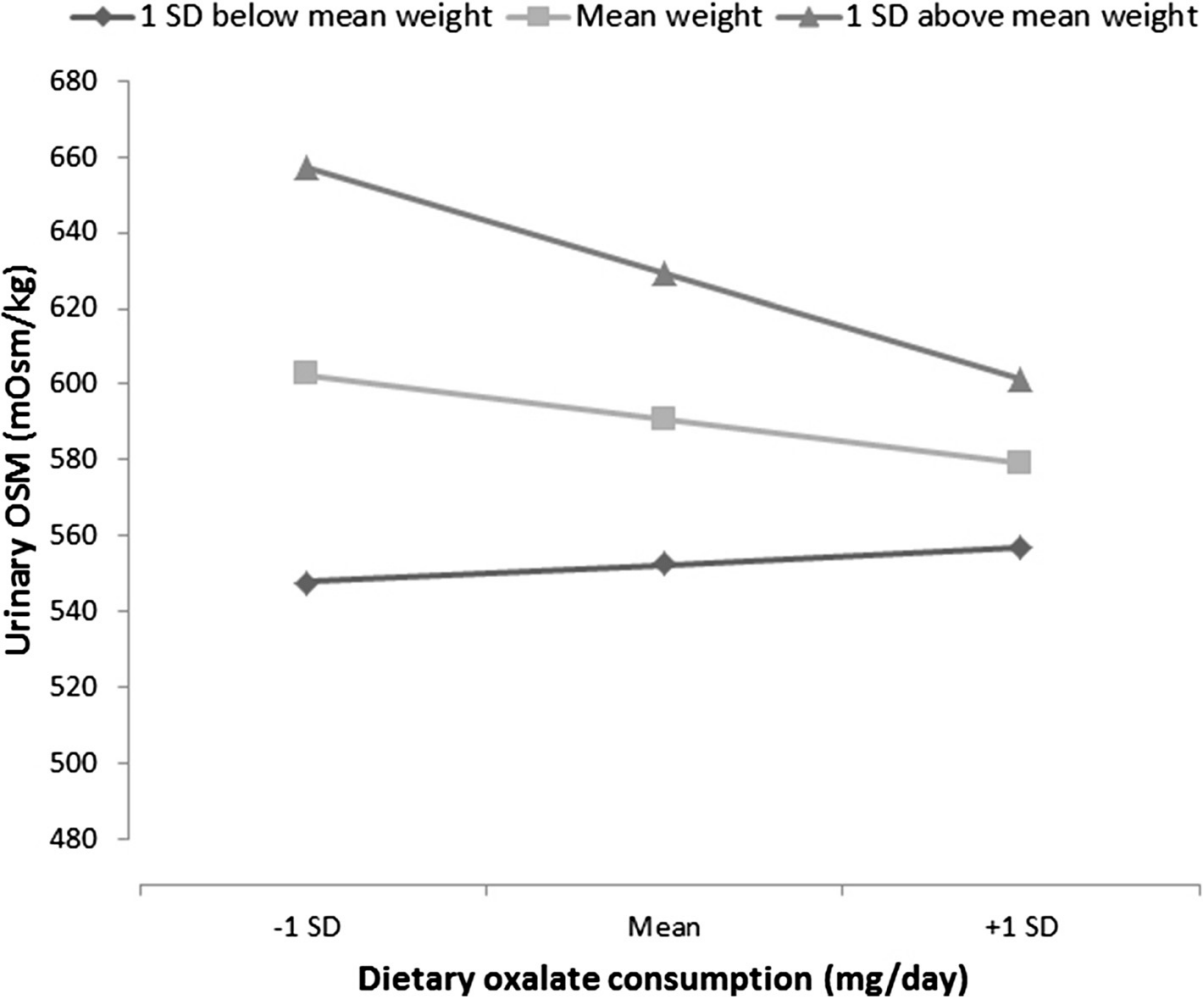
Effect of dietary oxalate on urine osmolality at different weights (*β* = −0.006, *p* = 0.04). Higher weight associated with greater change in urine osmolality on oxalate intake, suggesting increased dietary variation as an underlying factor

In the multivariable model not including interactions (Table 3), urine osmolality declined with age and water intake and remained higher in males than females, accounting for serum creatinine, weight, and dietary measures. Weight and animal protein intake were positive predictors of urine osmolality. The only significant interaction for urine osmolality was between weight and oxalate intake (*β* = −0.006, *p* = 0.04) (Fig. 4). Water intake was the only variable significantly associated with urine volume in a multivariable model that included age, sex, serum creatinine, and dietary sodium. There were no significant interactions with age or sex for these measures. The predictors of urine osmolality did not differ in a sensitivity analysis that included only participants known to be non-stone formers (data not shown).

**Table 3:** Multivariable associations for urine osmolality and volume

	Urine osmolality, mOsm/kg	Urine volume, mL/day
	β	β
Intercept	721.6***	1809.7***
Age, years	−4.79***	−2.13
Sex (male)	95.9***	117.8
Serum creatinine, mg/dL	−7.32	−298.3
Weight, kg	1.93***	
Dietary measures		
Animal protein g/day	1.45**	
Oxalate, mg/day	−0.072	
Sodium, mg/day	0.0076	−0.034
Water Intake, g	−0.058***	0.21***

Water intake includes water from food consumption

*β* beta estimate

*p value <0.05; **p value <0.01; ***p value <0.001

## Discussion

Urine concentration (and hence fluid intake and urine volume) is thought to be a common risk factor for urinary stones. The current study revealed several interesting demographic features that associate with urine concentration. On average, men excrete a greater number of milliosmoles per day than women at any given urine volume. Thus, men consistently have more concentrated urine (Fig. 3). Maximal urine osmolality also declines with age (Fig. 1). Overall, these associations may contribute to known epidemiologic trends in stone disease.

One striking observation was that urine osmolality was higher in males than females. This could contribute to the known higher incidence of kidney stones in men [4]. The sex difference in urine osmolality did not significantly interact with demographic features, despite males having significantly greater weight and animal protein and sodium intake as compared to females. Although females had a slightly higher water intake than males, no significant sex difference was found in urine volume (Table 1). This might reflect higher insensible losses in women as compared to men, since women had higher water intake and lower urine osmolality, but similar urine volume. In a study by Parks and colleagues [16], male stone formers had reduced urine volume and sodium excretion during summer months, while women maintained urine volume despite reductions in urine sodium, implying insensible sodium losses.

In this study, males excreted more osmoles per day than females (Table 1). This was composed of roughly equal proportions of electrolytes and urea. Thus, men appear to have higher protein as well as electrolyte intake. Despite the higher salt intake, water intake was lower in men and urine volumes nearly the same. Looked at another way, males excreted their daily osmole load in a smaller urine volume across the spectrum of osmole intake (Fig. 3). These observations implicate altered thirst and vasopressin action between the sexes. Perucca and colleagues [6] made a similar observation and suspected that men’s thirst/vasopressin system had higher threshold than those of women and that they drink proportionally less.

Previously, studies have suggested sex differences in vasopressin’s renal efficacy and a lower thirst in males [6, 7]. A study of almost 500 German children [17] found that girls had a lower urinary osmolality than boys and a relatively higher urine volume. Higher values for plasma and urinary vasopressin have also been reported in men compared to women, and this same sex differential has been observed in rats [18]. One in vivo animal study demonstrated intravenous infusion of 2.5 M NaCl for 60 min resulted in higher vasopressin plasma concentrations in male rats compared to female rats [19]. Similarly, in a human study [7], hypertonic saline infusion resulted in a greater plasma vasopressin concentration in response to changes in plasma osmolality among eight men compared to eight women. There was no difference in free water clearance, suggesting concurrent lower renal vasopressin sensitivity in men compared to women. Liu and colleagues [20] also demonstrated that female rats express significantly more renal vasopressin 2 receptor (V(2)R) mRNA and protein in their kidneys than males, physiologically resulting in greater sensitivity to V(2)R agonist administration. Overall, the ability to concentrate urine is dependent on vasopressin’s antidiuretic effect, which in turn is influenced by the effect of renal prostaglandins [21] on medullary blood flow [22]. Both physiologic effects appear to vary between men and women [23–25]. Thus, data from humans and animals both support sex difference in renal concentrating ability.

The kidney’s ability to maximally concentrate urine declines with age [26]. However, in our study, urine osmolality was independent of serum creatinine or eGFR in the main effect model. Previously, Rowe and colleagues [27] studied the effect of a 12-h period of dehydration and demonstrated a significant decrease in urine osmolality with advancing age independent of the age-related decline in creatinine clearance. Phillips and colleagues [28] found that healthy older men (mean age 71) had a deficit in thirst and water intake after 24 h of water deprivation compared to younger men (mean age 23). The older group had a greater increase in vasopressin levels, but a lower urine osmolality, suggesting renal response to vasopressin was reduced. This decreased sensitivity to vasopressin’s antidiuretic effect among older individuals could be related to structural differences in the aging kidney such as increased fibrosis and decreased parenchymal mass [28, 29]. Studies in the medulla of aged rats have also suggested a decrease in many key transport proteins that participate in urine concentrating ability (aquaporins, urea transporters, V2 receptor) with reduced response to water restriction and administration of supra-physiologic dose of desmopressin [26].

Several interesting trends with diet were observed. Weight was a positive predictor of urine osmolality and had a significant interaction with oxalate intake (Fig. 4), suggesting diet was an underlying factor. This observation implies that the balance of higher and lower type of oxalate foods varies depending on weight. Animal protein intake was associated with higher urine osmolality, likely due to the low water density and high protein content of meat, the metabolism of which produces urea. Interestingly, in a main effect model in which dietary sodium and water intake were omitted, oxalate intake was associated with higher urine volume and lower urine osmolality. Notably, oxalate is found in fruits and vegetables, but not meat, chicken, or fish. Thus, oxalate may serve as a proxy for fruit and vegetable ingestions, which in turn provide greater free water than animal protein sources. A low-oxalate diet is often recommended for preventing the recurrence of calcium-oxalate stones [3]. However, Taylor and colleagues [30] suggested that dietary oxalate was not a major risk factor for kidney stone formation. This group also examined retrospectively the impact of the diets similar to the Dietary Approaches to Stop Hypertension (DASH) program on stone formation [31]. Among men and women participants, those with a higher DASH score ingested more calcium and oxalate, but had reduced kidney stone risk. Higher DASH scores associated with higher urine volume and higher citrate which appeared to offset the higher urinary oxalate. Together, these data suggest some oxalate containing foods could reduce stone risk depending on the ratio between the water and oxalate content, but further studies are needed to evaluate this.

Our study has weaknesses, such as a lack of data on physical activity and non-renal water loss. Also, our participants were limited to white Americans of European descent and of relatively older age. We also examined a largely non-stone-forming population. However, studying non-stone formers allowed us to more precisely assess age and gender influences on urine chemistry without being confounded by changes in dietary habits initiated as the result of forming stones.

## Conclusion

This study revealed several interesting trends related to urinary osmolality and volume. In general, men excrete more osmoles per day than women, but for any given osmole load do so in less volume, and hence in a more concentrated manner. Urine osmolality also declines with age in both sexes. Data from this study and others support the hypothesis that there are sex differences in thirst and vasopressin action. Since low urine volume and high urine concentration is a common kidney stone risk factor, these observations could explain, in part, well-established patterns of stone risk by age and sex.

## Additional file

Additional file 1: Table S1. Bivariate associations for variables that did not pass stepwise linear model selection criteria. **Figure S1.** Analysis of biological sex on variability in urine osmolality (A) and urine volume (B). (DOCX 50 kb)

## Abbreviations

BMI: body mass index
CC: cystatin C
CKD: chronic kidney disease
DASH: Dietary Approaches to Stop Hypertension
DBP: diastolic blood pressure
eGFR: estimated glomerular filtration rate
eGFR_Cys_: estimated glomerular filtration rate from cystatin C
GDUL: Genetic Determinants of Urinary Lithogenicity
GENOA: Genetic Epidemiology Network of Arteriopathy
SBP: systolic blood pressure

## References

[1] Scales CD, Smith AC, Hanley JM, Saigal CS (2012). Prevalence of kidney stones in the United States. Eur Urol.

[2] Qaseem A, Dallas P, Forciea MA, Starkey M, Denberg TD (2014). Dietary and pharmacologic management to prevent recurrent nephrolithiasis in adults: a clinical practice guideline from the American College of Physicians. Ann Intern Med.

[3] Pearle MS, Goldfarb DS, Assimos DG, Curhan G, Denu-Ciocca CJ, Matlaga BR (2014). Medical management of kidney stones: AUA guideline. J Urol.

[4] Lieske JC, Rule AD, Krambeck AE, Williams JC, Bergstralh EJ, Mehta RA (2014). Stone composition as a function of age and sex. Clin J Am Soc Nephrol.

[5] Taylor EN, Stampfer MJ, Curhan GC (2005). Obesity, weight gain, and the risk of kidney stones. Jama.

[6] Perucca J, Bouby N, Valeix P, Bankir L (2007). Sex difference in urine concentration across differing ages, sodium intake, and level of kidney disease. Am J Physiol Regul Integr Comp Physiol.

[7] Stachenfeld NS, Splenser AE, Calzone WL, Taylor MP, Keefe DL (2001). Sex differences in osmotic regulation of AVP and renal sodium handling. J Appl Physiol (1985)..

[8] Daniels PR, Kardia SL, Hanis CL, Brown CA, Hutchinson R, Boerwinkle E (2004). Familial aggregation of hypertension treatment and control in the Genetic Epidemiology Network of Arteriopathy (GENOA) study. Am J Med.

[9] Rule AD, Bailey KR, Lieske JC, Peyser PA, Turner ST (2013). Estimating the glomerular filtration rate from serum creatinine is better than from cystatin C for evaluating risk factors associated with chronic kidney disease. Kidney Int.

[10] Kristal AR, Kolar AS, Fisher JL, Plascak JJ, Stumbo PJ, Weiss R (2014). Evaluation of web-based, self-administered, graphical food frequency questionnaire. J Acad Nutr Diet.

[11] Hess B, Hasler-Strub U, Ackerman D, Jaeger P (1997). Metabolic evaluation of patients with recurrent idiopathic calcium nephrolithiasis. Nephrol Dial Transplant..

[12] Parks JH, Goldfisher E, Asplin JR, Coe FL (2002). A single 24-hour urine collection is inadequate for the medical evaluation of nephrolithiasis. J Urol.

[13] Wu W, Yang D, Tiselius HG, Ou L, Mai Z, Chen K (2015). Collection and storage of urine specimens for measurement of urolithiasis risk factors. Urology.

[14] Inker LA, Schmid CH, Tighiouart H, Eckfeldt JH, Feldman HI, Greene T (2012). Estimating glomerular filtration rate from serum creatinine and cystatin C. N Engl J Med.

[15] R Development Core Team (2008). A language and environment for statistical computing.

[16] Parks JH, Barsky R, Coe FL (2003). Gender differences in seasonal variation of urine stone risk factors. J Urol.

[17] Ebner A, Manz F (2002). Sex difference of urinary osmolality in German children. Am J Nephrol..

[18] Share L, Crofton JT, Ouchi Y (1988). Vasopressin: sexual dimorphism in secretion, cardiovascular actions and hypertension. Am J Med Sci.

[19] Ota M, Crofton JT, Liu H, Festavan G, Share L (1994). Increased plasma osmolality stimulates peripheral and central vasopressin release in male and female rats. Am J Physiol.

[20] Liu J, Sharma N, Zheng W, Ji H, Tam H, Wu X (2011). Sex differences in vasopressin V(2) receptor expression and vasopressin-induced antidiuresis. Am J Physiol Renal Physiol.

[21] Anderson RJ, Berl T, McDonald KD, Schrier RW (1975). Evidence for an in vivo antagonism between vasopressin and prostaglandin in the mammalian kidney. J Clin Invest.

[22] Nakanishi K, Mattson DL, Gross V, Roman RJ, Cowley AW (1995). Control of renal medullary blood flow by vasopressin V1 and V2 receptors. Am J Physiol.

[23] Wang YX, Crofton JT, Share L (1997). Sex differences in the cardiovascular and renal actions of vasopressin in conscious rats. Am J Physiol.

[24] Sullivan JC, Sasser JM, Pollock DM, Pollock JS (2005). Sexual dimorphism in renal production of prostanoids in spontaneously hypertensive rats. Hypertension.

[25] Parekh N, Zou AP, Jungling I, Endlich K, Sadowski J, Steinhausen M (1993). Sex differences in control of renal outer medullary circulation in rats: role of prostaglandins. Am J Physiol.

[26] Sands JM (2009). Urinary concentration and dilution in the aging kidney. Semin Nephrol.

[27] Rowe JW, Shock NW, DeFronzo RA (1976). The influence of age on the renal response to water deprivation in man. Nephron.

[28] Phillips PA, Rolls BJ, Ledingham JG, Forsling ML, Morton JJ, Crowe MJ (1984). Reduced thirst after water deprivation in healthy elderly men. N Engl J Med.

[29] Metcalfe W (2007). How does early chronic kidney disease progress? A background paper prepared for the UK Consensus Conference on early chronic kidney disease. Nephrol Dial Transplant..

[30] Taylor EN, Curhan GC (2007). Oxalate intake and the risk for nephrolithiasis. J Am Soc Nephrol.

[31] Taylor EN, Fung TT, Curhan GC (2009). DASH-style diet associates with reduced risk for kidney stones. J Am Soc Nephrol.

