# Supplementary material for: Key influence of sex on urine volume and osmolality
Source: Biol Sex Differ. 2016 Feb 9;7:12. doi: 10.1186/s13293-016-0063-0 (PMC4748596; doi:10.1186/s13293-016-0063-0)

**Supplemental table –Bivariate associations for variables that did not pass stepwise linear model selection criteria**

|  | **Urine Osmolality, mOsm/kg** | **Urine Volume, mL/d** |
| --- | --- | --- |
|  | β | β |
| BMI | 6.79^***^ | -1.15 |
| Smoker (yes) | -35.48 | 47.18 |
| Diabetes (yes) | 24.42 | 39.31 |
| SBP, mmHg | -1.54^***^ | -0.07 |
| DBP, mmHg | 0.31 | 2.53 |
| Blood Glucose, mg/dL | 0.95^**^ | 2.17 |
| eGFR_Cys_, ml/min/1.73m^2^ | 0.21 | 4.55^***^ |
| **Diuretic Use** |  |  |
| Loop (yes) | -66.68^*^ | 121.90 |
| Thiazide (yes) | 13.73 | -14.37 |
| **Dietary measures** |  |  |
| Calcium, mg/d | 0.00 | 0.12^*^ |
| Fructose, g/d | 0.17 | 2.12 |
| Total Protein, g/d | 0.84^***^ | 2.60^**^ |
| Sucrose, g/d | -0.61 | 1.45 |

β: beta estimate, BMI: body mass index, SBP: systolic blood pressure, DBP: diastolic blood pressure, eGFR_Cys_: estimated Glomerular Filtration Rate (cystatin calculation); *P-value<0.05; **P-value:<0.01; ***P-value:<0.001

**Supplemental figures - Analysis of biological sex on variability in urine osmolality (A) and urine volume (B)**

To compare the overall variability in urine osmolality and urine volume between the sexes, the distributions of osmolality were plotted separately by gender below. This demonstrates that variability in urine osmolality and urine volume are fairly similar between the sexes, and follow a fairly normal distribution in each. A Levine's test (Folded F) was also performed for the equality of variance in osmolality and urine volume. These tests were performed in the unrelated sample of GENOA participants that were used to do the forward selection modeling. The p-values for the Levine’s test of osmolality and urine volume were 0.1466 and 0.5154, respectively. Thus, there is no evidence that the variances are different between the sexes.

**A**


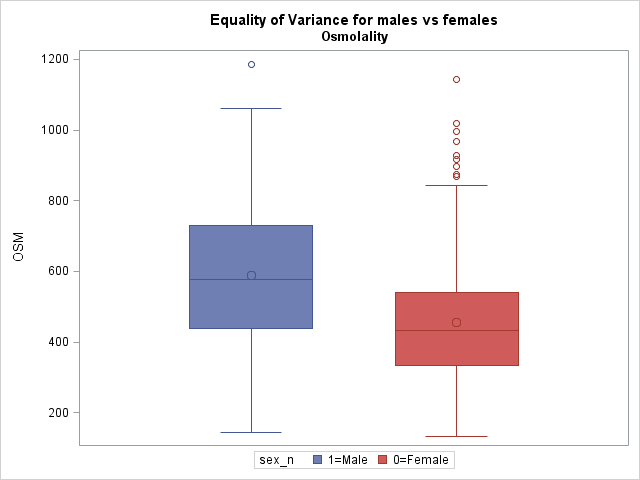


**B**


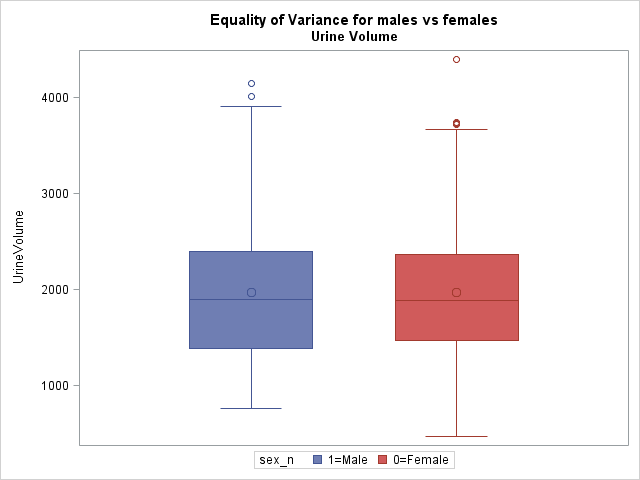

Supplement: Additional file 1: Table S1. — Bivariate associations for variables that did not pass stepwise linear model selection criteria. Figure S1. Analysis of biological sex on variability in urine osmolality (A) and urine volume (B). (DOCX 50 kb) [file 13293_2016_63_MOESM1_ESM.docx]
